# Advances in Red Blood Cells Research

**DOI:** 10.3390/cells13040359

**Published:** 2024-02-18

**Authors:** Anna Bogdanova, Lars Kaestner

**Affiliations:** 1 Red Blood Cell Group, Institute of Veterinary Physiology, University of Zurich, 8057 Zurich, Switzerland; 2 Theoretical Medicine and Biosciences, Campus of Saarland University Hospital, Saarland University, 66424 Homburg, Germany; 3 Dynamics of Fluids, Experimental Physics, Saarland University, 66123 Saarbrücken, Germany

## 1. Introduction

This Editorial ‘Advances in Red Blood Cell Research’ is the preface for the special issue with the same title which files 14 contributions listed in Table 1. This collection of articles compiles recent developments and ‘hot spots’ in red blood cell research. These cells inspired generations of scientists for centuries, and still harbor surprises such as the recent identification of the mechanosensitive ion channel Piezo1 as the Er-blood group-defining protein [1], or the association between the RH-blood group and the high-altitude acclimatization [2]. The majority of the papers within this special issue represent translational research, spanning from the investigation of the molecular mechanisms of pathologies (Table 1, #6, and #10) to the novel methodological approaches to diagnose diseases (Table 1, #1, #6, and #14), case (Table 1, #11) and epidemiological (Table 1, #3) studies, and transfusion medicine (Table 1, #13). Several articles focus on the factors influencing red blood cell production in vivo and in culture. These factors include hypoxia (Table 1, #5), immunophenotypic profile of precursor cells (Table 1, #12), their cytoskeleton and membrane lipid composition (Table 1, #10). In-depth studies of the rheological properties of red blood cells of humans and animals are presented (Table 1, #2, #4, #8, and #11). Finally, the need for an age-specific approach to estimate the causes of anemia in the elderly population is justified (Table 1, #3).

## 2. Red Blood Cells as Markers and Cause of Pathology: From Genetics to Functional Tests

Red blood cells are increasingly recognized as biomarkers, e.g., of the severity of the disease manifestation for red blood cell-related diseases, such as hereditary and acquired anemias (Table 1, #2, #3, and #6), but also for other pathologies that are not primarily linked to red blood cells, such as inflammation [3], cancer [4], diabetes [5,6] and cardiovascular disorders [7] (Table 1, #14). However, the tests that would establish a relation between the factors that define severity of the systemic pathological condition and the red blood cell properties are missing and common red blood cell indices, in particular the red blood cell distribution width, are currently used as such markers, which are not very specific. Recent studies reveal the promising potential for functional tests including red blood cell rheology, vital morphology and responses of these parameters to extreme conditions. This includes the response to swelling, shear or deoxygenation in judging the severity of the patients’ condition. Such functional tests are currently only available in a few clinical laboratories and the equipment to perform those under standardized conditions is limited. An example of using these tools to assess the disease state in sickle cell disease patients is presented by Boisson et al. (Table 1, #2).

The authors demonstrate that the oxygenscan parameters such as the ‘point of sickling’ are reflective of sickle cell disease genotype (hemoglobin mutation variant) and treatment (hydroxyurea or chronic red blood cell exchange). These oxygenscan parameters were proven to be associated with clinical severity of the sickle cell disease and sensitive to the responsiveness of the patients to therapy. Newest technological developments in which high throughput imaging and microfluidic devices are implicated allow detection of rheological responses of the individual cells to stress conditions such as shear or deoxygenation [8]. Testing of these new approaches in the clinical setting will show if they may match or even outperform ektacytometry which provides the mean values for deformability, membrane stability and hydration state for red blood cells in a given blood sample. One more article reports on the use of the osmoscan mode of the Lorrca Maxsis for the diagnosis of a compound hereditary anemia caused by a co-inheritance of beta-spectrin [9] and pyruvate kinase [10] gene mutations in a Spanish family (Table 1, #6). The prevalence of beta-spectrin-induced phenotype possibly caused by the pyruvate kinase variant in the manifestation of pathology was demonstrated.

Kuo et al. (Table 1, #4) investigated the properties of red blood cells with Gp.Mur a clinically relevant antigen of the MNS blood group system [11]. They found a decreased deformability and increased osmotic resistance in Gp.Mur red blood cells due to higher expression of Band3 protein and stronger Band3-aquaporin interaction, respectively.

An interesting observation reported for the feline patient with hereditary methemoglobinemia [12] with reduced function of cytochrome b5 reductase [13] due to two mutations in the corresponding gene suggests that accumulation of methemoglobin may increase deformability and reduce the stability of the red blood cell membrane (Table 1, #11).

One more report of this special issue brings ektacytometry closer to the veterinary clinical laboratory. In their study, Varga et al. (Table 1, #8) define the reference ranges for the Lorrca osmoscan test parameters for dogs, cats, sheep, rabbits, rats and mice. Earlier, a remarkable variance in red blood cell deformability was reported for several species using various techniques [14,15,16,17].

In a particular group of diseases known as the neuroacanthocytosis syndrome [18], patients carry a dedicated phenotype red blood cell shape, the acanthocytes (Table 1, #1). They were proposed as a diagnostic marker [19], although the relation of this peculiar red blood cell morphology to the mutations causing neuroacanthocytosis is still elusive.

In the special issue (Table 1, #1), the authors set the scene for a new physical explanation for the processes behind one of the oldest diagnostic tests used in clinical laboratories today, the erythrocyte sedimentation rate (ESR) [20]. The study demonstrates that the red blood cell aggregate formation alone is insufficient to describe the sedimentation rate. In contrast, a colloidal physics explanation, based on a percolating gel structure and consecutive collapse of the gel is much more appropriate to describe red blood cell sedimentation dynamics. Furthermore, it was found that for neuroacanthocytosis syndrome patients, the ESR is significantly longer than in controls, giving (for the first time) a useful threshold for a diagnostic interpretation of a slow ESR. In this paper (Table 1, #1) Darras et al. set the base for both, a deeper physical explanation [21,22] of the ESR and an application of the ESR in the context of neuroacanthocytosis investigations [23]. We expect further studies in particular addressing the question of differential diagnosis.

## 3. Findings on the In Vivo Erythropoiesis and In Vitro Red Blood Cell Culturing

Environmental stressors such as hypoxia, hyper- and hypothermia [24], and endurance and extreme sports practices [25,26,27] impact the numbers and properties of red blood cells.

Exploring the effects of these stressors on erythroid precursor cells, their properties, and processes driving their differentiation and proliferation during erythropoiesis (Table 1, #10, and #12) may help to improve conditions for the in vitro production of red blood cells of rare blood groups or those that may be used for transfusion for all patients. In vitro cultures of erythroid cells are normally maintained in incubators where atmospheric air is supplemented with 5% CO_2,_, i.e., at oxygen concentrations of around 20% while in the bone marrow pO_2_ is substantially lower [28]. Within this special issue (Table 1, #5), Simionato et al. investigated the impact of hypoxia on the in vitro erythropoiesis of CD34+ cells collected from the healthy donors at sea level (110 m, Heidelberg, Germany) and while under hypoxic conditions at the Jungfraujoch Research station (3450 m, Switzerland, [29]).

Today, ‘making one’s own red blood cells in a bioreactor’ is a realistic, although rather expensive option. Developing optimal methods for preserving red blood cells obtained from donors and storage in blood banks is of decisive importance for decreasing the costs and for successful blood transfusions. The need for improvement and novel approaches to assess the quality of the blood-derived products used for transfusion is recognized at the state and international level [30,31], and the potential for lab-on-the-chip technology and artificial intelligence has recently been revealed [32]. In line with this, the effect of the sex of the donor and the recipient in defining the efficacy of transfusion was investigated in one of the studies in this issue (Table 1, #13). In their work, Laengst et al. (Table 1, #13) experimented with transfusion simulations and observed sex-dependent hemolytic differences with lower hemolysis in the presence of female-derived plasma.

A further study (Table 1, #12) characterized the kinetics of expression of a panel of immunotypic markers occurring during in vitro differentiation of the human CD34+ precursor cells. This panel of markers may be used to detect stage-specific anomalies in patients with dyserythropoietic anemia and monitor the treatment’s success. The article also provides insights into the role of NDS1 histone lysine methyltransferase during the differentiation of erythroid precursor cells. Patients with severe hemolytic or dyserythropoietic anemia are in constant need of blood transfusion therapy.

## 4. On the Red Blood Cell Morphology and the Factors in Control of Their Fractionation in Percoll Density Gradients

Fractionation of red blood cells in Percoll density gradients is used for decades to obtain the red blood cell fractions enriched with young, senescent and mature cells. However, the in-depth understanding of the behavior of red blood cells during fractionation is incomplete, as stated earlier [33], and revealed in the study of Maurer et al. (Table 1, #7). The Percoll nanoparticles mediate red blood cell aggregation. As a result, the fractionation on the self-forming Percoll density gradient does not ‘order’ the cells according to the single cell density, but reflects more likely the average density of the red blood cells in an aggregate, while individual cells in the aggregate may have a broad range of densities.

Furthermore, the shapes of red blood cells adapt when they are resuspended in autologous plasma or serum (Table 1, #9). In general, numerous studies investigate and classify red blood cell shapes in 2D (e.g., [34,35]) or in 3D [36]. However, the thickness of the cells is rarely quantified. In his work, Thomas Fischer determined the ratio of thickness across the dimple region to the thickness of the rim to be 0.55 and 0.6 in plasma and serum, respectively. Consideration of these data in models of red blood cells will likely improve the models in terms of agreement with observations.

## 5. Summary and Outlook

Taken together, the contributions included in this special issue (Table 1) represent a fair cross-section of the areas of interest in modern red blood cell research. We are glad to declare this issue completed and are looking forward to a follow-up collection to illustrate further developments in the field.

## Figures and Tables

**Table 1:** Overview of titles that are part of the Special Topic.

Number	Type	Field	Title
1	research article	diagnostics, rare diseases	Acanthocyte Sedimentation Rate as a Diagnostic Biomarker for Neuroacanthocytosis Syndromes: Experimental Evidence and Physical Justification
2	research article	genetics, diagnostics	Effects of Genotypes and Treatment on Oxygenscan Parameters in Sickle Cell Disease
3	research article	epidemiology	Causes of Anemia in Polish Older Population—Results from the PolSenior Study
4	research article	rare diseases	Different Involvement of Band 3 in Red Cell Deformability and Osmotic Fragility—A Comparative GP.Mur Erythrocyte Study
5	research article	erythropoiesis	In Vitro Erythropoiesis at Different pO_2_ Induces Adaptations That Are Independent of Prior Systemic Exposure to Hypoxia
6	research article	rare diseases, diagnostics	Concomitant Hereditary Spherocytosis and Pyruvate Kinase Deficiency in a Spanish Family with Chronic Hemolytic Anemia: Contribution of Laser Ektacytometry to Clinical Diagnosis
7	research article	basic science	Continuous Percoll Gradient Centrifugation of Erythrocytes—Explanation of Cellular Bands and Compromised Age Separation
8	research article	comparative physiology	Interspecies Diversity of Osmotic Gradient Deformability of Red Blood Cells in Human and Seven Vertebrate Animal Species
9	research article	basic science	The Shape of Human Red Blood Cells Suspended in Autologous Plasma and Serum
10	research article *	erythropoiesis	Membrane Properties of Human Induced Pluripotent Stem Cell-Derived Cultured Red Blood Cells
11	research article	veterinary medicine, rare diseases	Methemoglobinemia, Increased Deformability and Reduced Membrane Stability of Red Blood Cells in a Cat with a *CYB5R3* Splice Defect
12	research article	erythropoiesis	Analysis of Immunophenotypic Changes during Ex Vivo Human Erythropoiesis and Its Application in the Study of Normal and Defective Erythropoiesis
13	research article	transfusion medicine	The Effect of the Donor’s and Recipient’s Sex on Red Blood Cells Evaluated Using Transfusion Simulations
14	review	diagnostics	Diagnostic Value and Prognostic Significance of Nucleated Red Blood Cells (NRBCs) in Selected Medical Conditions

* Editors’ Choice.

## References

[1] Crew V.K., Tilley L.A., Satchwell T.J., AlSubhi S.A., Jones B., Spring F.A., Walser P.J., Freire C.M., Murciano N., Rotordam M.G. (2022). Missense Mutations in PIEZO1, Encoding the Piezo1 Mechanosensor Protein, Define the Er Red Blood Cell Antigens. Blood.

[2] D’Alessandro A., Earley E.J., Nemkov T., Stephenson D., Dzieciatkowska M., Hansen K.C., Minetti G., Champigneulle B., Stauffer E., Pichon A. (2024). Genetic Polymorphisms and Expression of Rhesus Blood Group RHCE Are Associated with 2,3-Bisphosphoglycerate in Humans at High Altitude. Proc. Natl. Acad. Sci. USA.

[3] Marques O., Weiss G., Muckenthaler M.U. (2022). The Role of Iron in Chronic Inflammatory Diseases: From Mechanisms to Treatment Options in Anemia of Inflammation. Blood.

[4] Pfeifhofer-Obermair C., Tymoszuk P., Petzer V., Weiss G., Nairz M. (2018). Iron in the Tumor Microenvironment—Connecting the Dots. Front. Oncol..

[5] Association A.D. (2018). 2. Classification and Diagnosis of Diabetes: Standards of Medical Care in Diabetes—2019. Diabetes Care.

[6] Mzimela N.C., Sosibo A.M., Ngubane P.S., Khathi A. (2023). The Changes in Red Blood Cell Indices That Occur in Pre-Diabetic Patients of All Ethnicities from the 25–45 Years of Age: A Protocol for a Systematic Review and Meta-Analysis. Methods Protoc..

[7] Heshmat-Ghahdarijani K., Fakhrolmobasheri M. (2022). Is Red Cell Distribution Width a Reliable Marker for Cardiovascular Diseases? A Narrative Review. Cardiol. Rev..

[8] Recktenwald S.M., Lopes M.G.M., Peter S., Hof S., Simionato G., Peikert K., Hermann A., Danek A., van Bentum K., Eichler H. (2022). Erysense, a Lab-on-a-Chip-Based Point-of-Care Device to Evaluate Red Blood Cell Flow Properties with Multiple Clinical Applications. Front. Physiol..

[9] Karinch A.M., Zimmer W.E., Goodman S.R. (1990). The Identification and Sequence of the Actin-Binding Domain of Human Red Blood Cell Beta-Spectrin. J. Biol. Chem..

[10] Blume K.G., Hoffbauer R.W., Busch D., Arnold H., Löhr G.W. (1971). Purification and Properties of Pyruvate Kinase in Normal and in Pyruvate Kinase Deficient Human Red Blood Cells. Biochim. Biophys. Acta.

[11] Lamis R.J.S., Chiueh T.-S., Tsai C.-H., Lo H.-R., Wei S.-C., Chao Y.-C. (2021). Identification and Quantification of Anti-Gp.Mur Antibodies in Human Serum Using an Insect-Cell-Based System. Diagnostics.

[12] Scott E.M., Griffith I.V. (1959). The Enzymic Defect of Hereditary Methemoglobinemia: Diaphorase. Biochim. Biophys. Acta.

[13] Hultquist D.E., Passon P.G. (1971). Catalysis of Methaemoglobin Reduction by Erythrocyte Cytochrome B5 and Cytochrome B5 Reductase. Nat. N. Biol..

[14] Pesen T., Haydaroglu M., Capar S., Parlatan U., Unlu M.B. (2023). Comparison of the Human’s and Camel’s Red Blood Cell Deformability by Optical Tweezers and Raman Spectroscopy. Biochem. Biophys. Rep..

[15] McNamee A.P., Kuck L., Simmonds M.J. (2022). Bovine Erythrocytes Are Poor Surrogates for Human When Exposed to Sublethal Shear Stress. Int. J. Artif. Organs.

[16] Amin T.M., Sirs J.A. (1985). The Blood Rheology of Man and Various Animal Species. Q. J. Exp. Physiol..

[17] Smith J.E., Mohandas N., Shohet S.B. (1979). Variability in Erythrocyte Deformability among Various Mammals. Am. J. Physiol.-Heart Circ. Physiol..

[18] Walker R.H., Peikert K., Jung H.H., Hermann A., Danek A. (2023). Neuroacanthocytosis Syndromes: The Clinical Perspective. Contact.

[19] Storch A., Kornhass M., Schwarz J. (2005). Testing for Acanthocytosis. J. Neurol..

[20] Ernst E. (1990). Erythrocyte Sedimentation Rate. History and Importance Today. Acta Med. Austriaca.

[21] Darras A., Dasanna A.K., John T., Gompper G., Kaestner L., Fedosov D.A., Wagner C. (2022). Erythrocyte Sedimentation: Collapse of a High-Volume-Fraction Soft-Particle Gel. Phys. Rev. Lett..

[22] Dasanna A.K., Darras A., John T., Gompper G., Kaestner L., Wagner C., Fedosov D.A. (2022). Erythrocyte Sedimentation: Effect of Aggregation Energy on Gel Structure during Collapse. Phys. Rev. E.

[23] Rabe A., Kihm A., Darras A., Peikert K., Simionato G., Dasanna A.K., Glaß H., Geisel J., Quint S., Danek A. (2021). The Erythrocyte Sedimentation Rate and Its Relation to Cell Shape and Rigidity of Red Blood Cells from Chorea-Acanthocytosis Patients in an Off-Label Treatment with Dasatinib. Biomolecules.

[24] Kameneva M.V., Ündar A., Antaki J.F., Watach M.J., Calhoon J.H., Borovtez H.S. (1999). Decrease in Red Blood Cell Deformability Caused by Hypothermia, Hemodilution, and Mechanical Stress. ASAIO J..

[25] Carin R., Deglicourt G., Rezigue H., Martin M., Nougier C., Boisson C., Dargaud Y., Joly P., Renoux C., Connes P. (2023). Effects of a Maximal Exercise Followed by a Submaximal Exercise Performed in Normobaric Hypoxia (2500 m), on Blood Rheology, Red Blood Cell Senescence, and Coagulation in Well-Trained Cyclists. Metabolites.

[26] Szanto S., Mody T., Gyurcsik Z., Babjak L.B., Somogyi V., Barath B., Varga A., Matrai A.A., Nemeth N. (2021). Alterations of Selected Hemorheological and Metabolic Parameters Induced by Physical Activity in Untrained Men and Sportsmen. Metabolites.

[27] Nader E., Monedero D., Robert M., Skinner S., Stauffer E., Cibiel A., Germain M., Hugonnet J., Scheer A., Joly P. (2020). Impact of a 10 Km Running Trial on Eryptosis, Red Blood Cell Rheology, and Electrophysiology in Endurance Trained Athletes: A Pilot Study. Eur. J. Appl. Physiol..

[28] Li C., Zhao R., Yang H., Ren L. (2023). Construction of Bone Hypoxic Microenvironment Based on Bone-on-a-Chip Platforms. Int. J. Mol. Sci..

[29] Klein M., Kaestner L., Bogdanova A.Y., Minetti G., Rudloff S., Lundby C., Makhro A., Seiler E., van Cromvoirt A., Fenk S. (2021). Absence of Neocytolysis in Humans Returning from a Three-Week High-Altitude Sojourn. Acta Physiol..

[30] European Commission, Directorate-General for Health and Food Safety (2022). Proposal for a REGULATION OF THE EUROPEAN PARLIAMENT AND OF THE COUNCIL on Standards of Quality and Safety for Substances of Human Origin Intended for Human Application and Repealing Directives 2002/98/EC and 2004/23/EC 2022.

[31] Isiksacan Z., D’Alessandro A., Wolf S.M., McKenna D.H., Tessier S.N., Kucukal E., Gokaltun A.A., William N., Sandlin R.D., Bischof J. (2023). Assessment of Stored Red Blood Cells through Lab-on-a-Chip Technologies for Precision Transfusion Medicine. Proc. Natl. Acad. Sci. USA.

[32] Lopes M.G.M., Recktenwald S.M., Simionato G., Eichler H., Wagner C., Quint S., Kaestner L. (2023). Big Data in Transfusion Medicine and Artificial Intelligence Analysis for Red Blood Cell Quality Control. Transfus. Med. Hemotherapy.

[33] Bogdanova A., Kaestner L. (2020). Early Career Scientists’ Guide to the Red Blood Cell—Don’t Panic!. Front. Physiol..

[34] Routt A.H., Yang N., Piety N.Z., Lu M., Shevkoplyas S.S. (2023). Deep Ensemble Learning Enables Highly Accurate Classification of Stored Red Blood Cell Morphology. Sci. Rep..

[35] Sadafi A., Bordukova M., Makhro A., Navab N., Bogdanova A., Marr C. (2023). RedTell: An AI Tool for Interpretable Analysis of Red Blood Cell Morphology. Front. Physiol..

[36] Simionato G., Hinkelmann K., Chachanidze R., Bianchi P., Fermo E., van Wijk R., Leonetti M., Wagner C., Kaestner L., Quint S. (2021). Red Blood Cell Phenotyping from 3D Confocal Images Using Artificial Neural Networks. PloS Comput. Biol..

